# Factors Associated with Height-Promoting Dietary Practices Among Japanese Preschool Children: A Web-Based Cross-Sectional Study

**DOI:** 10.3390/children13030391

**Published:** 2026-03-12

**Authors:** Kemal Sasaki, Tomomi Kobayashi, Yuki Tada, Yasuyo Wada, Tetsuji Yokoyama

**Affiliations:** 1 Department of Nutrition and Dietetics, Jissen Women’s University, Tokyo 191-8510, Japan; 2 Department of Food Science and Nutrition, Mukogawa Women’s University, Nishinomiya 663-8558, Japan; t_koba@mukogawa-u.ac.jp; 3 Department of Nutritional Science, Tokyo University of Agriculture, Tokyo 156-8502, Japan; y3tada@nodai.ac.jp; 4 Department of Health Promotion, National Institute of Public Health, Wako 351-0197, Japan; wada.y.aa@niph.go.jp (Y.W.); yokoyama.t.aa@niph.go.jp (T.Y.)

**Keywords:** short stature, health literacy, information seeking, dietary intake, mothers, preschool children

## Abstract

**Highlights:**

**What are the main findings?**

• Height-related dietary practices were associated with child age, history of short stature, history of food allergy, information-seeking, and shorter parental height.
• Milk and dairy products were the most commonly used foods for height promotion; however, few children met the intake frequency cutoffs across food groups.

**What are the implications of the main findings?**

• Selective, growth-focused dietary modifications may not be accompanied by broader improvements in diet across food groups.
• Guidance may be more effective when maternal concerns are acknowledged, and feasible and balanced eating patterns are supported.

**Abstract:**

**Background/Objectives:** Parents of preschool children may adopt dietary practices intended to promote height growth. However, the correlates of such practices in the general population remain unclear. **Methods:** This web-based cross-sectional study included 1362 mothers of Japanese children aged 3–5 years. Mothers were assigned to the height-related dietary practice group if they had ever used dietary practices to promote their child’s height growth; all other mothers were classified into the no-practice group. The questionnaire assessed child and parental characteristics, including anthropometric measurements, history of short stature (height < −2 SD at any time point), history of food allergy, maternal information-seeking behavior regarding child growth, and current dietary intake. Associations with height-related dietary practices were examined using a multiple logistic regression analysis. **Results:** In total, 531 mothers (39.0%) were classified into the practice group. Older child age, history of short stature, history of food allergy, and information-seeking behavior were positively associated with height-related dietary practices, whereas parental height was inversely associated. Milk and dairy products are the most frequently used foods for height promotion. Children in the practice group were more likely to meet the cutoffs for milk/dairy products and fish, but their overall attainment was low. In the analyses of retrospectively reported height SD score trajectories, no group-by-time interactions were observed. **Conclusions:** Height-related dietary practices were associated with growth-related concerns and parental characteristics rather than differential height gain over time. Health guidance may help parents adopt balanced diets that support overall nutrition and healthy growth rather than focusing on specific foods.

## 1. Introduction

Linear growth during early childhood is commonly monitored using standardized height measurements, which are longitudinally recorded and shared with caregivers through growth charts and routine health checkup systems [1,2]. Adequate nutrition during this period is widely recognized as an essential component of healthy growth and development [3,4], and several observational studies have reported associations between linear growth in children and the intake of specific food groups, including milk and dairy products and other animal-source foods [5,6]. However, evidence linking habitual intake of specific food groups in everyday settings with linear growth is mixed, with substantial heterogeneity across study designs and populations [7,8]. Parents often pay close attention to their children’s growth patterns and may develop concerns about height during early childhood [9]. Despite the widespread availability of growth-related information through routine health services, little is known about how parents of the general population interpret such information and translate growth-related concerns into concrete dietary practices in daily life.

Internationally, height approximately −2 standard deviations (SD) from age- and sex-specific reference values is widely used as an important threshold for identifying short stature (SS) and for further clinical evaluation or treatment [10,11]. In this study, we use the term “short stature” to refer to this individual-level height threshold in the context of child growth assessment and caregiver concerns, rather than the population-level undernutrition construct of “stunting.” In Japan, the Maternal and Child Health Handbook (MCHH) is issued to nearly all pregnant women during pregnancy registration and is commonly used by families during early childhood [12]. Repeated measurements of child height are routinely recorded during municipality-based infant and early childhood health checkups and are documented in the MCHH, enabling caregivers to track growth trajectories over time [12]. In addition to the MCHH, parents may access child health and parenting information through health professionals and other sources [13,14]. However, although growth information is readily available, it remains unclear how parents in the general population interpret deviations from reference values, and whether or how these interpretations are associated with the adoption of dietary practices specifically intended to support child height growth.

Based on this background, the present cross-sectional study aimed to describe the dietary practices that mothers implement with the explicit intention of supporting their preschool child’s height growth and to examine factors associated with the presence of such practices in the general population. We hypothesized that height-related dietary practices are more strongly associated with parental characteristics and growth-related concerns than with children’s actual height gain over time. In addition, we examined the types of foods targeted for this purpose, and whether the current usual intake frequencies of major food groups differed according to the implementation of height-related dietary practices.

## 2. Materials and Methods

### 2.1. Study Design and Participants

This web-based cross-sectional survey was conducted among mothers of preschool children in Japan. On 22 January 2024, an online research company (NTT Com Online Marketing Solutions Corporation, Shinagawa, Tokyo, Japan) invited 1400 mothers registered on their online panel to participate in the survey. Eligible participants were mothers residing in Japan who were living with a child aged three to five years and who were native Japanese speakers. To reduce the potential bias related to professional knowledge and childcare circumstances, mothers working in health-related professions were excluded. Mothers with multiple births and those whose children were born preterm (gestational age, <37 weeks) were also excluded. These exclusion criteria were assessed using maternal self-report in the survey questionnaire. To ensure data quality, responses judged to be invalid according to predefined criteria were excluded from the analysis. Invalid responses were defined as follows: (1) straight-line responses to the questions on the frequency of intake of different food groups (*n* = 2), (2) logically contradictory answers (*n* = 1), (3) biologically implausible values for anthropometric measurements, indicating probable data entry or reporting errors (*n* = 6), and (4) clearly inconsistent dates for the anthropometric measurements (*n* = 21). Additionally, mothers who reported that their child had a history of congenital heart disease, endocrine disorders, or other conditions that could have a non-negligible impact on growth were excluded (*n* = 8). Mothers in health-related professions were identified from self-reported professional qualifications, and information on multiple birth status, gestational age, and child conditions affecting growth used for exclusion was also obtained by maternal self-report; no external verification was performed. After these exclusions, 1362 mothers were included in the final analysis.

### 2.2. Measures

#### 2.2.1. Child and Parental Characteristics

Information on the child and parental characteristics was collected using a self-administered structured questionnaire. Child characteristics included age, sex, gestational age at birth, use of daycare or preschool services, history of food allergy, and experience of medical consultation for SS. Maternal characteristics included age, highest educational attainment, employment status, smoking status, and alcohol consumption. The height and weight of both the mother and father were self-reported. Parental height was treated as a continuous variable and used as a proxy for the genetic potential of height in the analyses. Maternal health literacy was assessed using the Japanese version of the HLS-Q12 [15,16]. Responses were converted to a 0–50 health literacy index score using the standard formula (index = [mean − 1] × [50/3]), with higher scores indicating higher health literacy.

#### 2.2.2. Anthropometric Measurements

Mothers reported their child’s height and weight at birth, municipality-based infant and early childhood health checkups, and the most recent measurement. To improve the accuracy of reporting, mothers were instructed to respond by referring to records documented in the MCHH that contain records of childbirth and health checkups for children at 4, 18, and 36 months. SD values for anthropometric measurements were derived from the national survey data of Japanese children [17,18]. SD score (SDS) was calculated using the following formula: SDS = (x_i_ − x_s_)/SD, where x_i_ represents the individual measurement and x_s_ and SD represent the age- and sex-specific mean and SD from the reference data.

#### 2.2.3. Height-Related Dietary Practices

Experience of food-related practices intended to support a child’s height growth was assessed by asking, “Have you ever actively encouraged your child to eat specific foods to help increase your child’s height?” Mothers who answered “yes” were classified into the height-related dietary practice group (practice group), whereas those who answered “no” were classified into the no-height-related dietary practice group (no-practice group). Therefore, the practice group included mothers who had implemented such practices at any time in the past, regardless of the timing, duration, continuity, or intensity of practice. This broad definition was used to capture the presence of height-related dietary practices at the population level; however, it may combine heterogeneous behaviors and should be interpreted accordingly. Among mothers in the practice group, specific food groups targeted for height-related dietary practices were assessed by asking respondents to select all applicable items from a predefined list, including cereals, meat, fish, soy products, eggs, vegetables, fruits, milk, dairy products, and calcium-fortified foods. In addition, the use of dietary supplements explicitly intended to promote a child’s height growth was assessed, but was not included in the primary analyses, which focused on food-based dietary practices.

#### 2.2.4. Current Dietary Intake Frequency

Current dietary intake frequency, reflecting usual intake over the past month at the time of the survey, was assessed using a brief food frequency questionnaire developed and validated for Japanese preschool children [19,20]. This questionnaire was developed as a practical tool for use by health professionals to assess usual food-group intake patterns in preschool children. Therefore, it assesses intake frequency rather than portion size and was not intended to directly estimate nutrient intake or overall diet quality. The mothers were asked to report the usual frequency of intake of major food groups, including cereals, meat, fish, soy products, eggs, vegetables, fruits, milk, and dairy products. This assessment was analyzed separately from the implementation of height-related dietary practices (Section 2.2.3). Calcium-fortified foods were assessed only as foods used for height promotion, and were not included in the food frequency questionnaire used to assess current usual intake. For the primary analyses, the intake frequency for each food group was dichotomized using pre-specified cutoffs adopted from our validated questionnaire (Section 2.3).

#### 2.2.5. Information-Seeking Behavior

Mothers’ information-seeking behavior related to their child’s growth was assessed using a multiple-choice question asking whether they had ever obtained information about their child’s height or growth from a specific source. Response options included the MCHH, public health staff (such as public health nurses in municipalities), healthcare providers (e.g., physicians and nurses at medical facilities), childcare professionals (such as staff at nursery schools and kindergartens), family and friends, the Internet or social media, and an option indicating that no growth-related information had been sought [13,14].

For the analyses, information-seeking behavior was treated as a binary variable, classified as “yes” if mothers reported obtaining growth-related information from one or more sources and “no” if they reported not having obtained such information from any source. This variable was used to examine the association between exposure to growth-related information and the presence of height-related dietary practices. The present analysis was intended to capture overall engagement with growth-related information, rather than to compare the contribution of specific information sources. Accordingly, source-specific comparisons were presented descriptively, whereas the primary multivariable models used the binary variable to keep the main analysis parsimonious and to avoid multiple source-specific comparisons.

### 2.3. Definitions of Key Variables

SS was defined as a height of <−2 SD from age- and sex-specific reference values. A history of food allergy was defined as physician-diagnosed food allergy with the experience of eliminating the causative food(s). For each allergen, we summarized dietary avoidance as “ever avoided” (including participants who were currently avoiding the allergen) and “currently avoiding”; multiple allergens could be reported, and frequency was calculated using the total number of participants in each group as the denominator (Supplementary Table S1). Calcium-fortified foods, such as calcium-fortified milk or beverages, were defined as foods with added calcium, as indicated by product labeling. Dietary supplement use for height growth was defined as caregiver-reported use of dietary supplements with the explicit intention of supporting or promoting a child’s height growth.

For current dietary intake by food group, the cutoffs were prespecified based on our validated instrument, the brief food frequency questionnaire [19,20]. The following cutoffs were used to dichotomize intake frequency: 3–4 times/day for cereals; ≥2 times/day for milk and dairy products and vegetables; and ≥1 time/day for fruits, fish, meat, eggs, and soy products. A binary variable was created for each food group, indicating whether the child met the cutoff value. These dichotomized variables were used as behavioral indicators based on intake frequency cutoffs.

### 2.4. Statistical Analysis

The participants were classified into practice and no-practice groups. Comparisons of variables between the two groups were performed using univariate analyses, including Fisher’s exact test for categorical variables and unpaired *t*-test for continuous variables. For categorical comparisons, Fisher’s exact test was used consistently to provide exact inference without relying on large-sample approximations. The concordance between the use of selected foods for height promotion and current intake frequency meeting the cutoff is summarized in a four-cell table (Supplementary Table S3).

For multivariate analyses, two hierarchical models were defined for each analysis. Covariates were selected a priori based on the study objectives, prior literature, and conceptual relevance, rather than data-driven variable selection. Maternal health literacy was reported descriptively but not included in the primary multivariable models to preserve model parsimony, because maternal information-seeking behavior was considered more directly relevant to the primary research question. In both analyses, Model 1 was adjusted for child characteristics (child age [continuous per 1-year increase], child sex [reference: female], SDS of birth weight [continuous, per 1-point increase], and history of food allergy [reference: no]), and Model 2 was additionally adjusted for parental factors (maternal age [continuous, per 1-year increase], maternal and paternal height [continuous, per 10 cm increase], maternal educational attainment [reference: under high school], and maternal information-seeking behavior regarding child growth [reference: no]). All variables were simultaneously entered into each model.

Linear mixed-effects models were used to assess child height growth from birth to the health checkup at 36 months, according to the implementation of height-related dietary practices. The child height SDS was used as the dependent variable. Time (age at measurement) was treated as a within-subject factor and group (practice group vs. no-practice group) as a between-subject factor, and the interaction between group and time was included in all models. The time was modeled as categorical (assessment time points) rather than continuous to avoid imposing a linear growth assumption during early childhood. Linear mixed-effects models were fitted as an unadjusted model, Model 1, and Model 2 (as defined above); the history of SS was not included because it is derived from the outcome (height SDS) and would introduce redundancy and potential instability in the model. The results are presented as estimated marginal means with 95% confidence intervals.

Multiple logistic regression analyses were performed to explore the factors associated with the implementation of height-related dietary practices (dependent variable; reference category: no-practice group). Model 1 additionally included a history of SS (reference: no), and Model 2 added parental factors (as described above). Results are presented as adjusted odds ratios with 95% confidence intervals.

All variables used in the analyses were complete, and no missing data were observed. All statistical tests were two-sided, and a *p*-value of <0.05 was considered statistically significant. Statistical analyses were performed using IBM SPSS Statistics (ver. 30 (IBM Corp., Armonk, NY, USA)).

## 3. Results

### 3.1. Participant Characteristics

The final analysis sample included 1362 mothers, of whom 531 (39.0%) were classified into the practice group and 831 (61.0%) into the no-practice group (Table 1). Relative to the no-practice group, mothers in the practice group more frequently reported a history of SS in their child and a history of food allergy. A history of medical consultation for SS was also more common in the practice group; however, only one mother reported having received dietary guidance for height promotion. Among the children with a history of SS, 135 of 284 mothers (47.5%) were in the practice group, compared with 396 of 1078 mothers (36.7%) among those without a history of SS (calculated from Table 1). Additional descriptive information is provided in the Supplementary Tables.

### 3.2. Foods Used by Mothers to Promote Their Child’s Height

Table 2 summarizes the foods and products that mothers reported using with the intention of increasing their child’s height in the practice group. Multiple responses were obtained. Milk and dairy products were the most frequently reported items. Cereals and calcium-fortified foods are commonly used. Meat, soy products, fish, and vegetables were reported by a similar proportion of mothers, whereas fruits were selected less frequently. A small proportion of mothers reported using dietary supplements to increase their child’s height.

### 3.3. Factors Associated with the Implementation of Height-Related Dietary Practices

Table 3 presents the results of multiple logistic regression analyses examining the factors associated with the implementation of height-related dietary practices. Then, the two hierarchical models were fitted. In Model 1, which included only child characteristics, child age, child sex, history of SS, and history of food allergy were associated with the implementation of height-related dietary practices, whereas birth weight was not. In Model 2, after additional adjustment for parental factors, child age, history of SS, history of food allergy, and maternal information-seeking behavior regarding child growth remained associated with the implementation of height-related dietary practices. In contrast, maternal and paternal height were inversely associated with the implementation of these practices. The association with child sex was attenuated and was no longer statistically significant in Model 2.

### 3.4. Changes in Child Height SDS

Table 4 shows the results of the linear mixed-effects models examining changes in child height SDS from birth to health checkup at 36 months according to the implementation of height-related dietary practices. In the unadjusted model, the practice group showed a lower height SDS than did the no-practice group across the observation period. However, the interaction between the group and time was not statistically significant. After adjustment for child characteristics in Model 1 and for both child and parental factors in Model 2, the difference in height SDS between the practice and no-practice groups was attenuated, and the group-by-time interaction was not statistically significant.

### 3.5. Current Dietary Intake Frequency

Table 5 summarizes the current dietary intake frequency of children according to the implementation of height-related dietary practices. Relative to the no-practice group, a higher proportion of children in the practice group met the cutoffs for the intake frequency of milk/dairy products and fish. Across all food groups, the proportion of children who met the cutoff values was limited in both groups. We also summarized the concordance between ever use for height promotion and current intake frequency of milk/dairy products and fish (Supplementary Table S3). Among mothers who reported using these foods for height promotion, 85/420 (20.2%) for milk/dairy products and 21/139 (15.1%) for fish met the cutoffs (Supplementary Table S3).

### 3.6. Information Sources Related to Child Growth

Table 6 summarizes the sources of information on child growth reported by mothers in the practice and no-practice groups. The MCHH was the most frequently used source in both groups. Compared to the no-practice group, a higher proportion of mothers in the practice group reported obtaining information from public health staff, healthcare providers, and family or friends. The proportions of childcare professionals and the internet as information sources were similar between the two groups.

## 4. Discussion

In this cross-sectional study, we examined the factors associated with the implementation of height-related dietary practices among mothers of young children. We found that implementation was associated with child age, history of SS, history of food allergy, parental height, and information-seeking behavior regarding the child’s growth; notably, shorter maternal and paternal height and active information-seeking were associated with a higher likelihood of implementing these practices. In secondary analyses of height SDS trajectories up to 36 months, we did not observe evidence that trajectories differed by practice status after adjustment. These findings are consistent with the interpretation that the observed practices were associated with parental background characteristics and growth-related concerns, rather than differences in observed growth trajectories.

Shorter maternal and paternal heights were independently associated with implementation of height-related dietary practices. This finding suggests that parental anthropometric characteristics may shape parents’ perceptions and concerns regarding their children’s growth. Previous studies among families of children with SS have reported that parents are concerned about their child’s current and future height and potential psychosocial consequences, and that these concerns influence parental decision-making regarding growth-related interventions [21,22]. Importantly, the association between parental height and height-related dietary practices remained after adjusting for child factors, indicating that this relationship was not solely explained by the child’s growth status. Given the well-established association between parental height and child height [23,24], height-related dietary practices may reflect parental background characteristics and expectations regarding child growth, independent of the measured growth indicators in this study.

In the present study, the association between child sex and implementation of height-related dietary practices observed in Model 1 was attenuated and no longer statistically significant after additional adjustment for parental factors. This pattern suggests that the apparent difference by sex may reflect parental background characteristics and related concerns rather than the child’s sex. A male predominance has been reported in clinical evaluations and referrals for SS, which has been discussed as a potential manifestation of sex-related bias in growth concerns and management [11,21]. In our study, the attenuation of the association after accounting for parental height and information-seeking behavior is consistent with the interpretation that parental factors may play a more central role than child sex in shaping height-related dietary practices.

A history of food allergies was associated with implementation of height-related dietary practices. This may reflect co-occurring dietary constraints or heightened parental concern about growth and nutrition rather than the effectiveness of specific practices [25,26]. However, detailed information on allergy severity and duration of elimination is not available, and this interpretation should be interpreted with caution. As prolonged elimination diets may influence both dietary practices and growth outcomes, residual confounding related to unmeasured aspects of food allergies cannot be excluded.

We assessed both foods mothers reported using with the intention of promoting height (Table 2) and their children’s current intake frequency (Table 5). Milk and dairy products are the most commonly reported foods used for height promotion. This may reflect both the easy availability of milk in daily meals and the common perception that milk supports height growth. Such perceptions have also been reported in other settings; qualitative research has described caregivers’ views that milk supports child height growth [27]. Consistently, a community-based study from Korea reported that parents of relatively short children were more likely to use milk as a complementary measure of growth than parents of non-short children [28]. Several observational studies have reported associations between milk intake and height in children; however, a systematic review concluded that evidence for a consistent effect on linear growth remains inconclusive [8]. Children in the practice group were more likely than those in the no-practice group to meet the cutoff for the current fish intake frequency. Because health literacy and information-seeking behavior may shape caregivers’ dietary choices, the higher achievement of fish in the practice group may partly reflect differences in exposure to health information. However, fish-specific knowledge was not assessed in this study and this interpretation remains speculative. When all food groups were considered, the proportion of children meeting the cutoff intake frequency was generally low. Concordance analyses (Supplementary Table S3) suggested that a substantial proportion of mothers who had ever used food for height promotion did not currently meet the cutoff intake frequency. Although portion sizes were not assessed, this pattern suggests that parents may focus on a limited set of foods they believe contribute to height growth while paying less attention to the overall dietary balance. In Japan, a higher frequency of meals, including staple, main, and side dishes, has been associated with a higher nutrient intake [29]. A lower frequency of balanced meals has also been linked to early adiposity rebound among preschool children [20]. These findings underscore the need for practical guidance that emphasizes overall diet quality and balance rather than single foods for parents who adopt dietary practices aimed at promoting child height.

In the linear mixed-effects model, children in the practice group had a lower height SDS in the unadjusted analysis, but this difference was reduced after covariate adjustment, and we did not observe evidence of a group-by-time interaction. These results do not support the differential height SDS trajectories according to the practice status in this study. Birth weight, child sex, and parental height are well-established determinants of subsequent height growth, and inadequate control of these factors may exaggerate apparent associations with postnatal exposure [23,24,30,31]. The adjusted results suggest that the unadjusted between-group difference likely reflects differences in baseline child and parental characteristics rather than the effects of dietary practices. This null finding should also be interpreted cautiously because practice status captured ever implementation and did not reflect the timing, duration, continuity, or intensity of the practices.

In this study, the mothers of children with a history of SS were more likely to report height-related dietary practices. This finding suggests that perceived deviations in a child’s growth pattern may prompt parents to consider dietary modification a practical response in daily life, even in the absence of medical intervention. At the same time, it is noteworthy that even among mothers whose children had a history of height < −2 SD, only 47.5% reported implementing height-related dietary practices. This suggests variability in caregiver responses and decision-making when faced with concerns regarding short stature. Some parents may view dietary modification as a feasible and acceptable approach, whereas others may choose alternative strategies or may not regard dietary changes as necessary. These findings highlight the heterogeneity in parental interpretations of growth concerns and how such concerns are translated into everyday behaviors, underscoring that height-related dietary practices represent one of several possible responses to perceived SS rather than a uniform parental reaction.

Child’s age was positively associated with the implementation of height-related dietary practices. One possible interpretation is that parents of older children have had a longer period during which concerns about growth may emerge, and dietary approaches may be attempted. Rather than reflecting age-specific effects alone, this association may reflect cumulative exposure to growth-related information and experience over time. Maternal information-seeking behavior regarding child growth (defined as having sought growth-related information from at least one source) was also more common among mothers who reported height-related dietary practices. In particular, mothers in the practice group more frequently reported using interpersonal information sources such as public health staff, healthcare providers, and family or friends, whereas the use of MCHH was similarly high in both groups. Given that the MCHH is universally distributed and widely used in Japan as a foundational source of child health information [11], its use may not distinguish mothers who adopt height-related dietary practices from those who do not. Taken together, these findings suggest that height-related dietary practices and maternal information-seeking behavior may co-occur in the broader context of parental engagement with child growth and support-seeking. However, the cross-sectional design limits inferences about the directionality between information-seeking and the adoption of dietary practices.

From a practical perspective, these findings suggest that height-related dietary practices may reflect parental concerns about growth, rather than objective differences in growth trajectories. Growth-related counseling during routine child health checkups and widely used, universally available resources, such as the MCHH, could acknowledge such concerns while emphasizing feasible, balanced eating patterns that support overall nutritional adequacy rather than focusing on single foods.

This study has several limitations that should be considered when interpreting the findings. First, owing to its cross-sectional design, causal relationships cannot be inferred for the observed associations between the variables and height-related dietary practices. Because information-seeking behavior was assessed cross-sectionally as a lifetime experience, the temporal order between growth concerns, information-seeking, and the adoption of dietary practices could not be determined. Second, study variables were based on maternal self-report in the questionnaire and may be subject to recall and reporting biases. This includes child height and dietary intake, parental height and weight, and some eligibility/exclusion criteria, which may have introduced measurement error and some misclassification of eligibility. In addition, food allergy variable may not capture avoidance practices without a formal diagnosis, which could also influence dietary practices. Third, participants were recruited through an online survey, and mothers who chose to participate may have had a greater interest in child growth or nutrition than the general population. Therefore, selection bias cannot be excluded and the prevalence of height-related dietary practices may have been overestimated. Fourth, practice status was based on ever implementation of food-based practices. Because this measure did not capture timing, duration, continuity, or intensity, between-group comparisons should be interpreted cautiously. Finally, dietary intake was assessed in terms of food group frequency, rather than portion size or nutrient intake, and the continuity or intensity of dietary practices over time was not evaluated. Because practice status captured ever implementation, some caregivers may not have maintained these practices at the time current intake was assessed (Supplementary Table S3), which may have resulted in exposure misclassification and could have affected the observed between-group differences in current intake. Additionally, although supplement use intended to promote height was assessed, it was not included in the primary analyses. Thus, we could not evaluate how supplement use may have contributed to the overall nutrient intake in this sample. Therefore, the extent to which reported practices translate into meaningful differences in nutrient intake or growth-related outcomes remains unclear.

## 5. Conclusions

This study found that, among mothers of preschool children in Japan, the implementation of dietary practices intended to promote height growth was positively associated with the child’s age, history of SS, history of food allergy, and maternal engagement with growth-related information, and inversely associated with maternal and paternal height. Milk and dairy products were the most frequently used foods in these practices; however, the proportion meeting the current intake frequency cutoff was low. These findings suggest the importance of providing balanced, evidence-informed guidance for parents who adopt dietary approaches aimed at promoting child height. The null finding in the height SDS trajectory analysis should be interpreted cautiously, because the practice variable captured ever implementation and did not include information on the timing, duration, continuity, or intensity of the practices. Future longitudinal studies are needed to clarify temporal relationships and potential impacts on growth.

## Figures and Tables

**Table 1:** Characteristics of children and mothers according to height-related dietary practices.

	Total (*n* = 1362)		Practice Group (*n* = 531)		No-Practice Group (*n* = 831)	
**Child** **characteristics**						
Age (years)	4.2 ± 0.8		4.2 ± 0.8		4.1 ± 0.8	
Sex, boys	670	(49.2)	281	(53.2)	389	(47.1)
Gestational age (days)	276 ± 8		276 ± 8		276 ± 8	
Birth height (SD score)	−0.006 ± 1.063		−0.091 ± 1.076		0.049 ± 1.052	
Birth weight (SD score)	0.124 ± 0.873		0.072 ± 0.852		0.157 ± 0.885	
Current height (SD score)	−0.107 ± 1.153		−0.237 ± 1.164		−0.024 ± 1.138	
Current weight (SD score)	0.073 ± 0.911		−0.006 ± 0.939		0.124 ± 0.889	
History of short stature, yes	284	(20.9)	135	(25.4)	149	(17.9)
Consultation for short stature, yes	34	(2.5)	22	(4.1)	12	(1.4)
History of food allergy, yes	172	(12.6)	85	(16.0)	87	(10.5)
Attendance at nursery school or kindergarten	1273	(93.5)	504	(94.9)	769	(92.5)
**Parental characteristics**						
Maternal age (years)	34.6 ± 5.0		34.4 ± 4.9		34.7 ± 5.0	
Maternal height (cm)	158.3 ± 5.6		157.6 ± 5.4		158.8 ± 5.6	
Paternal height (cm)	172.0 ± 6.0		171.3 ± 5.9		172.5 ± 6.1	
Maternal body mass index (kg/m^2^)	21.1 ± 3.3		21.0 ± 3.2		21.1 ± 3.4	
Paternal body mass index (kg/m^2^)	23.3 ± 3.4		23.1 ± 3.3		23.3 ± 3.4	
Maternal ever smoking	270	(19.8)	104	(19.6)	166	(20.0)
Maternal smoking during pregnancy	30	(2.2)	9	(1.7)	21	(2.5)
Maternal ever alcohol consumption	998	(73.3)	383	(72.1)	615	(74.0)
Maternal alcohol consumption during pregnancy	22	(1.6)	7	(1.3)	15	(1.8)
Maternal educational attainment ≥ junior college	1023	(75.1)	398	(75.0)	625	(75.2)
Maternal health literacy (score of HLS-Q12)	27.8 ± 8.4		28.4 ± 8.7		27.4 ± 8.1	
Maternal employment status, employed	854	(62.7)	337	(63.5)	517	(62.2)

Data were collected anonymously and participants were informed that their responses would be used solely for research purposes. Values are presented as mean ± SD or *n* (%). Practice group: mothers who reported implementing dietary practices specifically to promote their child’s height growth. No-practice group: mothers who reported no such practices. Short stature was defined as a height of <−2 SD at any time point.

**Table 2:** Foods and products used in dietary practices intended to promote height growth.

	Mothers in the Practice Group (*n* = 531)	
Cereals	155	(29.2)
Fish	139	(26.2)
Meat	142	(26.7)
Eggs	127	(23.9)
Soy products	140	(26.4)
Vegetables	139	(26.2)
Fruits	76	(14.3)
Milk and dairy products	420	(79.1)
Calcium-fortified foods	157	(29.6)
Dietary supplements	26	(4.9)

Values are presented as *n* (%). Multiple answers were allowed.

**Table 3:** Factors associated with the implementation of height-related dietary practices.

	Model 1			Model 2		
	OR	95% CI	*p*-Value	OR	95% CI	*p*-Value
Child age	1.17	[1.01–1.35]	0.038	1.19	[1.02–1.39]	0.023
Child sex	1.30	[1.04–1.63]	0.020	1.25	[1.00–1.58]	0.054
Birth weight	0.90	[0.79–1.03]	0.124	0.95	[0.83–1.09]	0.491
History of short stature	1.55	[1.18–2.04]	0.002	1.38	[1.04–1.84]	0.024
Food allergy	1.61	[1.16–2.23]	0.004	1.51	[1.08–2.12]	0.016
Maternal age				0.99	[0.96–1.01]	0.290
Maternal height				0.67	[0.55–0.83]	<0.001
Paternal height				0.72	[0.60–0.88]	0.001
Maternal education				1.02	[0.78–1.33]	0.908
Information-seeking behavior regarding child growth				2.44	[1.91–3.12]	<0.001

Adjusted odds ratios (ORs) and 95% confidence intervals (CIs) were estimated using multiple logistic regression analyses. The dependent variable was the presence of height-related dietary practices (reference: no height-related dietary practice). Model 1 included child age (per 1-year increase), child sex (reference: female), birth weight (per 1-point increase in the SD score), history of short stature (reference: no), and history of food allergy (reference: no). Model 2 additionally included maternal and paternal height (per 10 cm increase), maternal education (reference: high school or below), and information-seeking behavior regarding child growth (reference: no).

**Table 4:** Height-SD from birth to 36 months.

	Birth	4 Months	18 Months	36 Months	*P* Group	*P* Time	*P* Group × Time
**Unadjusted**					<0.001	<0.001	0.687
Practice group	−0.091	−0.729	−0.612	−0.409			
	[−0.202–0.02]	[−0.842–−0.616]	[−0.733–−0.491]	[−0.525–−0.292]			
No-practice group	0.049	−0.517	−0.368	−0.186			
	[−0.04–0.137]	[−0.608–−0.426]	[−0.466–−0.271]	[−0.279–−0.092]			
Mean difference	−0.140	−0.212	−0.243	−0.223			
	[−0.282–0.002]	[−0.358–−0.067]	[−0.399–−0.088]	[−0.372–−0.073]			
**Model 1**							
Practice group	−0.112	−0.749	−0.628	−0.421	<0.001	<0.001	0.718
	[−0.224–0.001]	[−0.864–−0.635]	[−0.75–−0.506]	[−0.538–−0.303]			
No-practice group	−0.003	−0.570	−0.419	−0.239			
	[−0.101–0.094]	[−0.669–−0.47]	[−0.524–−0.313]	[−0.341–−0.138]			
Mean difference	−0.109	−0.180	−0.209	−0.181			
	[−0.241–0.024]	[−0.316–−0.044]	[−0.356–−0.063]	[−0.322–−0.041]			
**Model 2**							
Practice group	−0.067	−0.706	−0.580	−0.377	0.017	<0.001	0.730
	[−0.18–0.046]	[−0.821–−0.591]	[−0.703–−0.457]	[−0.496–−0.259]			
No-practice group	−0.023	−0.591	−0.437	−0.257			
	[−0.121–0.075]	[−0.692–−0.49]	[−0.544–−0.331]	[−0.359–−0.154]			
Mean difference	−0.044	−0.115	−0.142	−0.121			
	[−0.175–0.086]	[−0.249–0.019]	[−0.287–0.003]	[−0.259–0.018]			

Linear repeated-measures mixed model for the change in height-SD score from birth to 36 months. Values are presented as the estimated mean and 95% confidence interval. The mean difference is the value of the practice group minus that of the no-practice group. Model 1 included child age (per 1-year increase), child sex (reference: female), birth weight (per 1-point increase in the SD score), and history of food allergy (reference: no). Model 2 additionally included maternal and paternal height (per 10 cm increase), maternal education (reference: high school or below), and information-seeking behavior regarding child growth (reference: no).

**Table 5:** Proportion of children meeting frequency-based cutoffs for current food-group intake.

		Practice Group		No-Practice Group		*p*-Value
		(*n* = 531)		(*n* = 831)		
Cereals	Meets	357	(67.2)	538	(64.7)	0.350
	Does not meet	174	(32.8)	293	(35.3)	
Fish	Meets	35	(6.6)	33	(4.0)	0.040
	Does not meet	496	(93.4)	798	(96.0)	
Meat	Meets	116	(21.8)	177	(21.3)	0.839
	Does not meet	415	(78.2)	654	(78.7)	
Eggs	Meets	59	(11.1)	104	(12.5)	0.494
	Does not meet	472	(88.9)	727	(87.5)	
Soy products	Meets	59	(11.1)	73	(8.8)	0.160
	Does not meet	472	(88.9)	758	(91.2)	
Vegetables	Meets	155	(29.2)	216	(26.0)	0.212
	Does not meet	376	(70.8)	615	(74.0)	
Fruits	Meets	96	(18.1)	180	(21.7)	0.112
	Does not meet	435	(81.9)	651	(78.3)	
Milk and dairy products	Meets	93	(17.5)	88	(10.6)	<0.001
	Does not meet	438	(82.5)	743	(89.4)	

Values are presented as *n* (%). The cutoffs for intake frequency were defined as follows: 3–4 times/day for cereals; ≥2 times/day for milk and dairy products and vegetables; and ≥1 time/day for fruits, fish, meat, eggs, and soy products. “Meets” indicates meeting the cutoff for intake frequency. *p*-values were obtained using Fisher’s exact test.

**Table 6:** Sources of information on child growth.

		Practice Group		No-Practice Group		*p*-Value
		(*n* = 531)		(*n* = 831)		
Any source	Used	396	(74.6)	456	(54.9)	<0.001
	Not used	135	(25.4)	375	(45.1)	
Sources of information						
MCHH	Used	393	(74.0)	580	(69.8)	0.097
	Not used	138	(26.0)	251	(30.2)	
Public health staff	Used	212	(39.9)	264	(31.8)	0.002
	Not used	319	(60.1)	567	(68.2)	
Healthcare providers	Used	147	(27.7)	188	(22.6)	0.039
	Not used	384	(72.3)	643	(77.4)	
Childcare professionals	Used	72	(13.6)	89	(10.7)	0.122
	Not used	459	(86.4)	742	(89.3)	
Family or friends	Used	88	(16.6)	76	(9.1)	<0.001
	Not used	443	(83.4)	755	(90.9)	
Internet	Used	158	(29.8)	208	(25.0)	0.060
	Not used	373	(70.2)	623	(75.0)	

Abbreviation used: MCHH, Maternal and Child Health Handbook. Multiple responses were provided. Values are presented as *n* (%). *p*-values were obtained using Fisher’s exact test.

## Data Availability

The data presented in thisstudy are available on request from the corresponding author due to ethical reasons.

## Supplementary Materials

The following supporting information can be downloaded at: https://www.mdpi.com/article/10.3390/children13030391/s1, Table S1: Major food allergens: ever and current dietary avoidance by group; Table S2: Current food intake frequency for each food group; Table S3: Concordance between ever use for height promotion and current intake frequency meeting the cutoffs of selected foods (milk/dairy products and fish).

## Abbreviations

The following abbreviations are used in this manuscript:

MCHH	Maternal and Child Health Handbook
SD	Standard deviation
SDS	Standard deviation score
SS	Short stature
